# Ab Interno Trabeculectomy

**DOI:** 10.4103/0974-9233.71585

**Published:** 2010

**Authors:** Mina B. Pantcheva, Malik Y. Kahook

**Affiliations:** Rocky Mountain Lions Eye Institute, Department of Ophthalmology, University of Colorado Denver, Denver, CO 80045, USA

**Keywords:** Trabectome, Trabecular Meshwork, Trabeculectomy

## Abstract

Anterior chamber drainage angle surgery, namely trabeculotomy and goniotomy, has been commonly utilized in children for many years. Its’ reported success has ranged between 68% and 100% in infants and young children with congenital glaucoma. However, the long-term success of these procedures has been limited in adults presumably due to the formation of anterior synechiae (AS) in the postoperative phase. Recently, ab interno trabeculectomy with the Trabectome™ has emerged as a novel surgical approach to effectively and selectively remove and ablate the trabecular meshwork and the inner wall of the Schlemm’s canal in an attempt to avoid AS formation or other forms of wound healing with resultant closure of the cleft. This procedure seems to have an appealing safety profile with respect to early hypotony or infection if compared to trabeculectomy or glaucoma drainage device implantation. This might be advantageous in some of the impoverish regions of the Middle East and Africa where patients experience difficulties keeping up with their postoperative visits. It is important to note that no randomized trial comparing the Trabectome to other glaucoma procedures appears to have been published to date. Trabectome surgery is not a panacea, however, and it is associated with early postoperative intraocular pressure spikes that may require additional glaucoma surgery as well as a high incidence of hyphema. Reported results show that postoperative intraocular pressure (IOP) remains, at best, in the mid-teen range making it undesirable in patients with low-target IOP goals. A major advantage of Trabectome surgery is that it does not preclude further glaucoma surgery involving the conjunctiva, such as a trabeculectomy or drainage device implantation. As prospective randomized long-term clinical data become available, we will be better positioned to elucidate the exact role of this technique in the glaucoma surgical armamentarium.

## INTRODUCTION

The current gold standard for surgical treatment of glaucoma is trabeculectomy with adjunctive 5-fluouracil (5-FU) or mitomycin C (MMC), which bypasses the normal outflow channels by creating a direct opening into the episcleral space across the sclera.^1^ The long-term success of this procedure depends largely on the formation of a filtration bleb. It is also associated with complications such as hypotony maculopathy, bleb leaks, late blebitis, and bleb-related endophthalmitis.^2^,^3^

Glaucoma drainage devices have become a more commonly performed procedure in the last decade. Three-year data from the tube versus trabeculectomy study showed similar intraocular pressure (IOP) reduction and need for supplemental medical therapy in the two arms.^4^ Glaucoma drainage devices, however, are certainly not without complications such as conjunctival erosion, tube migration, motility disorders, tube obstruction, corneal decompensation, plate encapsulation, and late failure.^5^ Furthermore, if a tube fails the most viable options are another tube shunt or laser cilioablation. In contrast, for eyes failing a trabeculectomy, bleb needle revision may restore function or another trabeculectomy adjacent to the old site may be attempted before proceeding to a tube shunt.

Recently, there has been more interest in creating bleb-free and implant-free surgeries. Anterior segment studies indicated that the pressure-sensitive outflow occurs through the trabecular meshwork (TM), the canal of Schlemm (SC), and the collector channels. The main resistance to outflow of aqueous is presumed to be the juxtacanalicular connective tissue and the inner wall of Schlemm’s (SCIW) canal.^6^,^7^ This article discusses ab interno trabeculectomy using the Trabectome™, one of the new procedures, that aims to selectively remove the TM and SCIW while leaving the rest of the outflow system (outer wall of Schlemm’s canal, collector channels, and aqueous veins) relatively intact.

## AB INTERNO TRABECULECTOMY

The Trabectome™ (NeoMedix Inc., Tustin, CA, USA) device consists of a disposable handpiece tip (19.5-gauge) that will fit through a 1.6mm corneal incision. The handpiece is connected to a console with irrigation and aspiration and also to a simple electrocautery generator. The foot pedal controls the irrigation, aspiration, and electrocautery ablation via a stepwise foot control similar to a phacoemulsification system. The tip of the handpiece is specially designed with an insulated footplate and is pointed for ease of insertion through the TM into SC. The role of the footplate is critical in that it allows for the lifting of TM tissue while putting it on slight tension, and also allows for positioning the tissue for maximal discharge effect during electrocautery ablation while protecting the underlying tissues for preserving normal aqueous outflow. The footplate is coated with proprietary multilayered polymer, which provides exceptional thermal stability, mechanical strength, biocompatibility, and chemical resistance in laboratory testing. The aspiration port is in close proximity (approximately 0.3 mm) to the cautery electrode, and serves to remove debris during ablation. The irrigation is 3 mm from the surgical site and serves the dual purpose of keeping the eye pressurized and further dissipating heat energy. Although the irrigation and aspiration system role is important, the high-frequency electrocautery generator system is the pivotal point of this technology. The generator is a modified 800 EU unit from Aaron/Bovie (St. Petersburg, FL, USA), and operates at a frequency of 550 kHz with adjustable power setting in 0.1-W increment up to 10 W (recommended range 0.5–1.5 W). The target tissue is disrupted and disintegrated by applying heat energy in bursts with a high-peak power and low duty cycle. This ablation approach equates to high-energy bursts, which are bunched into small increments with comparably long-time intervals in between. As a result, disruption and disintegration of tissue is achieved rather than a thermal-cooking effect such as that seen in traditional cautery of blood vessels.^8^,^9^

The patient’s head is rotated opposite the eye-receiving treatment. Various anesthetic methods could be utilized, including retrobulbar, peribulbar, and sub-Tenon injection of 0.75% bupivacaine/2% lidocaine mixture and/or use of intracameral 1% preservative-free lidocaine. A near limbal 1.6-mm temporal corneal incision is made parallel to the iris, and viscoelastic (usually Ocucoat) is injected to inflate and stabilize the anterior chamber. Care is taken to avoid bubbles that can obscure the view of the angle. The Trabectome™ handpiece is advanced nasally across the anterior chamber with the infusion on. A modified Swan-Jacobs gonioscopy lens is used to visualize the target TM nasally as the instrument tip is advanced across the anterior chamber. The tip of the footplate is inserted through the TM into SC. A foot switch activates the aspiration and electro-surgical elements that ablates and removes the strip of TM and SCIW as the surgeon slowly advances the instrument along the meshwork in a clockwise and then counterclockwise direction using the insertion site as a fulcrum. A strip of TM and SCIW spanning 80°–100° is ablated and removed under direct gonioscopic visualization. Intraoperative reflux of blood through the resulting cleft is desirable in this procedure and confirms appropriate ab interno “unroofing” of Schlemm’s canal.

## CLINICAL RESULTS

In the initial clinical report of the procedure, IOP reduction of about 38% was achieved at the 6-month follow-up (*n* = 25). The number of adjunctive medications decreased from 1.2 ± 0.6 among preoperative patients on medications (*n* = 34) to 0.4 ± 0.6 among all patients at 6 months (*n* = 25). Blood reflux occurred in all eyes on instrument withdrawal after angle surgery and cleared by slit-lamp examination at a mean of 6.4 ± 4.1 days postoperatively.^10^ In a subsequent larger series of patients (*n* = 101) with follow up extended up to 30 months, the overall success rate, defined as IOP lower than 21 mmHg with or without medication and no subsequent glaucoma surgery, was determined to be 84%.^11^ Furthermore, a very low incidence of early hypotony and loss of vision in excess of two lines was reported with this procedure (<1% for both parameters).

Filippopoulos and Rhee published a review reporting an interim analysis of an ongoing prospective multicenter study evaluating the efficacy and safety of this novel procedure. The clinical outcome of 679 consecutive patients undergoing ab interno trabeculectomy with the Trabectome™ with a maximum follow-up of up to 52 months showed an average reduction in IOP of 29% at 6 months follow-up (*n* = 106), 34% at 12 months follow-up (*n* = 65), and 30% at 24 months follow-up (*n* = 30). This level of pressure reduction (30%) was sustained in the few patients who reached 48 months follow-up as of October 2007 (*n* = 13). In addition, a reduction in the number of glaucoma medications by about two medications was documented, which peaked at 10 months follow-up and remained stable thereafter. The most common indication for surgery in this cohort was primary open angle glaucoma (73%), followed by pseudoexfoliation (9%), pigment dispersion (3%), and uveitic glaucoma (2%). Very few patients (*n* = 7, 1%) experienced early transient hypotony (IOP < 5 mmHg) at the first postoperative day. The most common and the only clinically significant complication was a spike in IOP (IOP > 21 mmHg) in the early postoperative period (19.7%). The cumulative incidence of a subsequent glaucoma surgical procedure was 8% for this cohort with the overwhelming majority (85%) of surgeries being performed within 6 months after the Trabectome procedure, and 67% of the treating physicians preferring trabeculectomy as the second procedure. In 30% of the cases, the Trabectome™ procedure was combined with cataract extraction.^12^ Francis *et al*. reported the short-term results of combined phacoemulsification and ab interno trabeculectomy with Trabectome™ (*n* = 304). In most cases, the ab interno trabeculectomy was performed first, followed by cataract extraction by phacoemulsification.^13^ Success (i.e., 20% or greater drop in IOP or decrease in glaucoma medications without need for additional medications or glaucoma procedures, including laser trabeculoplasty) was 78% at 6 months (*n* = 106) and 64% at 12 months (*n* = 34). Nine patients (3%) required secondary glaucoma procedures (seven trabeculectomy, one aqueous tube shunt, and one selective laser trabeculoplasty). No patient had a decrease in Snellen visual acuity of two or more lines. An IOP spike of 10 mmHg or greater occurred in 8.6% of patients 1 day postoperatively and in 2.0% by 1 week. There were no serious complications such as those seen after trabeculectomy with MMC (e.g., sustained hypotony, choroidal effusion or hemorrhage, aqueous misdirection, infection, bleb formation, or wound leaks).

## CONCLUSIONS

Ab interno trabeculectomy seems to be a promising alternative surgical approach to lowering IOP when attempting to halt or slow down progression of IOP-induced glaucomatous optic neuropathy. It appears to be easy to perform, reproducible, with a low incidence of early postoperative hypotony (0–1%), and short-term adequate IOP control. Using the Trabectome™ involves added cost for both the console and the disposable handpieces needed for each procedure. This should be weighed against the potential benefits of avoiding a bleb postoperatively, and the complications that can come along with ab externo filtration surgery. The incidence of postoperative infection as a result of the procedure should be comparable with modern phacoemulsification (0 events so far in 679 cases).^12^ Furthermore, the conjunctiva remains undisturbed during this procedure, allowing conventional glaucoma surgery like trabeculectomy or drainage implant to remain available to the patient who is in need of better IOP control. The most common (60%) complication of the Trabectome™ procedure is transient hyphema/microhyphema as a result of intraoperative or postoperative blood reflux from Schlemm’s canal, which rarely persists past 1 week after the procedure.^10^ However, the Trabectome™ procedure cannot be expected to lower IOP any lower than episcleral venous pressure once the TM has been removed. Thus, it may not be suitable for patients with end-stage optic nerve cupping who are in need of lower IOP s (lower than mid-teens). It is unclear how results might be influenced when more tissue is removed compared to current limitations obtained through one corneal incision using a straight probe. The data regarding its use in other forms of glaucoma such as pediatric or uveitic remain sparse and require further study. We await long-term data to become available as well as randomized studies against trabeculectomy or drainage implants to clarify the role for ab interno trabeculectomy with Trabectome™ in the management of our patients with open-angle glaucoma.
